# Complete genome sequence of the plant-associated *Serratia plymuthica* strain AS13

**DOI:** 10.4056/sigs.2966299

**Published:** 2012-09-26

**Authors:** Saraswoti Neupane, Roger D. Finlay, Nikos C. Kyrpides, Lynne Goodwin, Sadhna Alström, Susan Lucas, Miriam Land, James Han, Alla Lapidus, Jan-Fang Cheng, David Bruce, Sam Pitluck, Lin Peters, Galina Ovchinnikova, Brittany Held, Cliff Han, John C. Detter, Roxanne Tapia, Loren Hauser, Natalia Ivanova, Ioanna Pagani, Tanja Woyke, Hans-Peter Klenk, Nils Högberg

**Affiliations:** 1 Department of Forest Mycology and Pathology, Swedish University of Agricultural Sciences, Uppsala, Sweden; 2 DOE Joint Genome Institute, Walnut Creek, California, USA,; 3 Los Alamos National Laboratory, Bioscience Division, Los Alamos, New Mexico, USA,; 4 Oak Ridge National Laboratory, Oak Ridge, Tennessee, USA; 5 Leibniz Institute DSMZ – German Collection of Microorganisms and Cell Cultures, Braunschweig, Germany

**Keywords:** Gram-negative, non-sporulating, motile, plant-associated, chemoorganotrophic, *Enterobacteriaceae*

## Abstract

*Serratia plymuthica* AS13 is a plant-associated *Gammaproteobacteria*, isolated from rapeseed roots. It is of special interest because of its ability to inhibit fungal pathogens of rapeseed and to promote plant growth. The complete genome of *S. plymuthica* AS13 consists of a 5,442,549 bp circular chromosome. The chromosome contains 4,951 protein-coding genes, 87 tRNA genes and 7 rRNA operons. This genome was sequenced as part of the project entitled “Genomics of four rapeseed plant growth promoting bacteria with antagonistic effect on plant pathogens” within the 2010 DOE-JGI Community Sequencing Program (CSP2010).

## Introduction

The members of the genus *Serratia* are widely distributed in nature. They are commonly found in soil, water, plants, insects, and other animals including humans [1]. The genus includes biologically and ecologically diverse species – from those beneficial to economically important plants, to pathogenic species that are harmful to humans. The plant-associated species comprise both endophytes and free living taxa, such as *S. proteamaculans, S. plymuthica, S. liquefaciens* and *S. grimesii*. Most of them are of interest because of their ability to promote plant growth and inhibit plant pathogenic fungi [2-6].

There are currently 16 validly named *Serratia* species. However, there are several unidentified plant-associated *Serratia* strains that have an impact on agriculture by stimulating plant growth and/or inhibiting soil borne plant pathogens [3]. *S. plymuthica* AS13 was isolated from rapeseed roots from Uppsala, Sweden. Our interest in *S. plymuthica* AS13 is due to its ability to stimulate rapeseed plant growth and to inhibit soil borne fungal pathogens such as *Verticillium dahlia* and *Rhizoctonia solani* [6]. Here we present a description of the complete genome of *S. plymuthica* AS13 and its annotation.

## Classification and features

A representative sequence of the 16S rRNA gene of *S. plymuthica* AS13 was compared with the most recently released GenBank databases using NCBI BLAST [7] under default settings. It showed that the strain AS13 shares 99-100% similarity with the genus *Serratia*. When considering high-scoring segment pairs (HSPs) from the best 250 hits, the most frequent matches were several unspecified *Serratia* strains (17.2%) with maximum identity of 97-100%, while *S. plymuthica* (5.2%) had maximum identity of 97-100%, *S. proteamaculans* (4.8%) maximum identity of 97-99%, *S. marcescens* (4.8%) maximum identity of 96-97% and also different *Rahnella* strains (7%) maximum identity of 97-98%.

**Figure 1:**
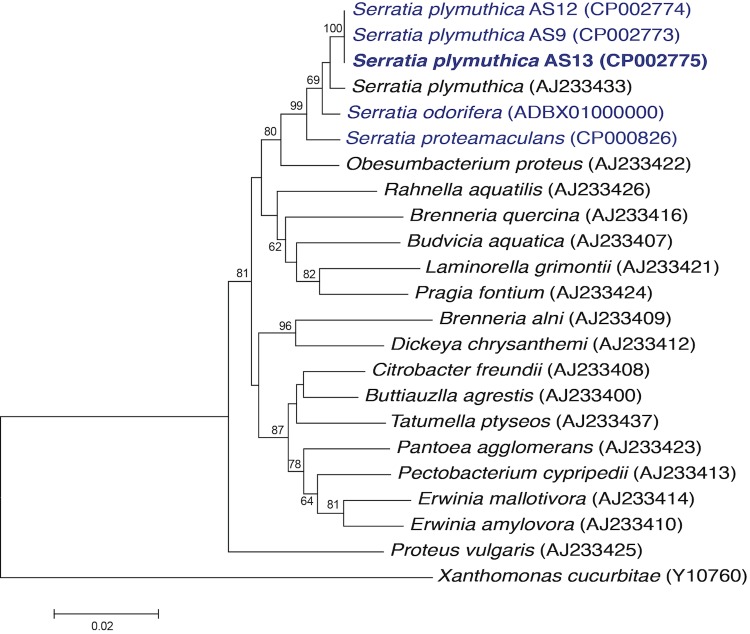
Phylogenetic tree highlighting the position of *S. plymuthica* AS13 in relation to other genera within the family *Enterobacteriaceae,* based on 1,472 characters of the 16S rRNA gene sequence aligned in ClustalW2 [8]. The tree was constructed under the maximum likelihood criterion using MEGA5 software [9] and rooted with *Xanthomonas cucurbitae* (a member of the *Xanthomonadaceae* family). The branches are scaled based on the expected number of substitutions per site. The numbers above branches are support values from 1,000 bootstrap replicates if larger than 60% [10]. The lineages shown in blue color are the genome sequences of bacterial strains that are registered in GOLD [11].

The phylogenetic relationship of *S. plymuthica* AS13 is shown in Figure 1 in a 16S rRNA based tree. All *Serratia* lineages clustered together and were distinct from other enterobacteria (except *Obesumbacterium proteus*). The tree also shows its very close relation with *S. plymuthica* strains AS9 and AS12, which was confirmed by digital DNA-DNA hybridization values [12] above 70% when compared with the (unpublished) draft genome sequence of the *S. plymuthica* type strain Breed K-7^T^ from a culture of DSM 4540, and when compared with the complete genome sequences of *S. plymuthica* AS9 [13] and *S. plymuthica* AS12 [14] using the GGDC web server [15].

**Figure 2:**
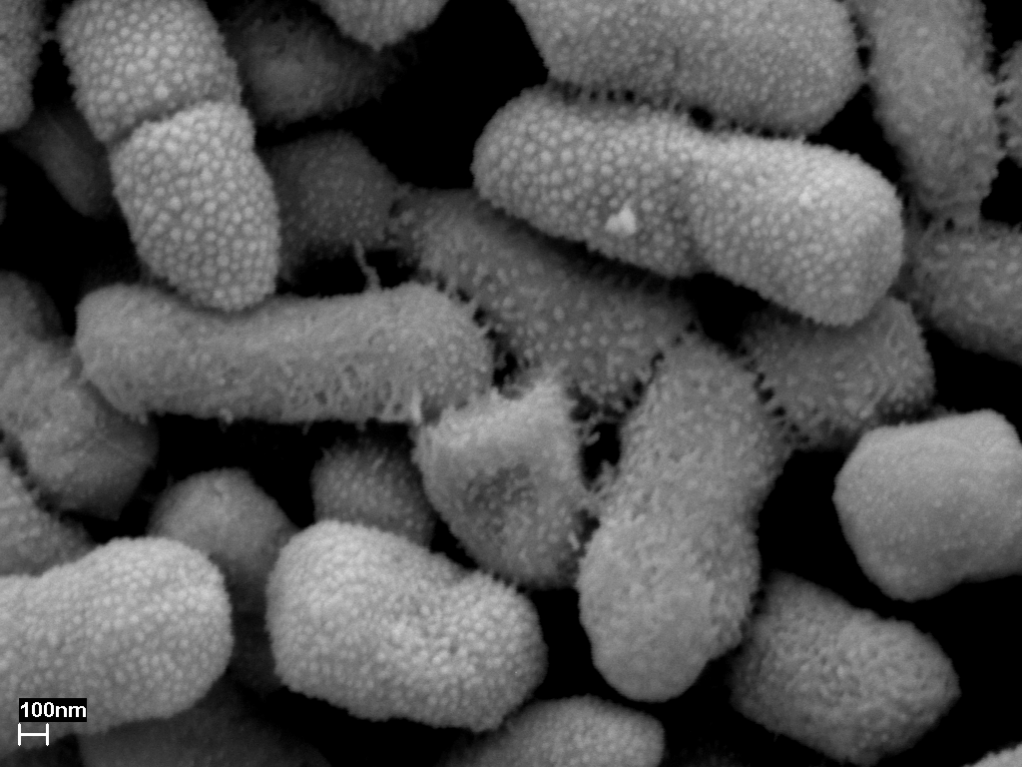
Scanning electron micrograph of *S. plymuthica* AS13

Strain AS13 is a rod shaped bacterium, 1-2 µm long, 0.5-0.7 µm wide (Figure 2 and Table 1), is Gram-negative, motile, and a member of the family *Enterobacteriaceae*. The bacterium is a facultative anaerobe and grows within the temperature range 4 °C - 40 °C and within a pH range of 4 - 10. It has chitinolytic, cellulolytic, proteolytic, and phospholytic activity [6] and can easily grow on different carbon sources such as glucose, cellobiose, succinate, mannitol, arabinose and inositol. It forms red to pink colored colonies that are 1-2 mm in diameter on potato dextrose agar at low temperature. The color of the bacterium depends on the growth substrate, temperature and pH of the culture medium [30]. The bacterium is deposited in the Culture Collection, University of Göteborg, Sweden (CCUG) as *S. plymuthica* AS13 (= CCUG 61398).

**Table 1:** Classification and general features of *S. plymuthica* AS13 according to the MIGS recommendations [16]

**MIGS ID**	**Property**	**Term**	**Evidence code**^a^
		Domain *Bacteria*	TAS [17]
		Phylum *Proteobacteria*	TAS [18]
		Class *Gammaproteobacteria*	TAS [19,20]
	Current classification	Order “*Enterobacteriales*”	TAS [21]
		Family *Enterobacteriaceae*	TAS [22-24]
		Genus *Serratia*	TAS [22,25,26]
		Species *Serratia plymuthica*	TAS [22,27]
		Strain AS13	IDA
	Gram stain	Negative	IDA
	Cell shape	Rod-shaped	IDA
	Motility	Motile	IDA
	Sporulation	Non-sporulating	IDA
	Temperature range	Mesophilic	IDA
	Optimum temperature	28°C	IDA
	Carbon source	Glucose, inositol, arabinose, succinate, sucrose, fructose	IDA
	Energy metabolism	Chemoorganotrophic	IDA
MIGS-6	Habitat	Rapeseed roots	IDA
MIGS-6.3	Salinity	Medium	IDA
MIGS-22	Oxygen	Facultative	IDA
MIGS-15	Biotic relationship	Plant associated	TAS [6]
MIGS-14	Pathogenicity	None	IDA
	Biosafety level	1	TAS [28]
MIGS-4	Geographic location	Uppsala, Sweden	NAS
MIGS-5	Sample collection time	Summer 1998	IDA
MIGS-4.1	Latitude	59.8	NAS
MIGS-4.2	Longitude	17.65	NAS
MIGS-4.3	Depth	0.1 m	NAS
MIGS-4.4	Altitude	24-25 m	NAS

a) Evidence codes - IDA: Inferred from Direct Assay; TAS: Traceable Author Statement (i.e., a direct report exists in the literature); NAS: Non-traceable Author Statement (i.e., not directly observed for the living, isolated sample, but based on a generally accepted property for the species, or anecdotal evidence). These evidence codes are from the Gene Ontology project [29]. If the evidence code is IDA, then the property should have been directly observed, for the purpose of this specific publication, for a live isolate by one of the authors, or an expert or reputable institution mentioned in the acknowledgements.

### Chemotaxonomy

Little is known about the chemotaxonomy of *S. plymuthica* AS13. Fatty acid methyl ester (FAME) analysis showed the main fatty acid in strain AS13 comprises C_16:0_ (25.27%), C_16:1ω7c_ (15.41%), C_18:1ω7c_ (18.17%), C_14:0_ (5.21%), C_17:0_ cyclo (18.53%), along with other minor fatty acid components. Previously it has been shown that *Serratia* spp. contain a mixture of C_14:0_, C_16:0_, C_16:1_ and C_18:1+2_ fatty acids in which 50-80% of the total fatty acid in the cell is C_14:0_ and other fatty acids are less than 3% each [31]. This is consistent with the fact that C_14:0_ fatty acid is characteristic of the family *Enterobacteriaceae*.

### Genome sequencing information

*S. plymuthica* AS13, a bacterial strain isolated from rapeseed roots was selected for sequencing on the basis of its biocontrol activity against fungal pathogens of rapeseed and its plant growth promoting ability. The genome project is deposited in the Genomes On Line Database [11] (GOLD ID = Gc01776) and the complete genome sequence is deposited in GenBank (INSDC ID = CP002775). Sequencing, finishing and annotation were performed by the DOE Joint Genome Institute (JGI). A summary of the project information is shown in Table 2 and its association with MIGS identifiers.

**Table 2:** Genome sequencing project information

**MIGS ID**	**Property**	**Term**
MIGS-31	Finishing quality	Finished
MIGS-28	Libraries used	Three libraries: one 454 standard library, one paired end 454 library (9.0 kb insert size) and one Illumina library)
MIGS-29	Sequencing platforms	Illumina GAii, 454 GS FLX Titanium
MIGS-31.2	Fold coverage	262.2 × Illumina, 8.7 × pyrosequencing
MIGS-30	Assemblers	Newbler version 2.3, Velvet 1.0.13, phrap version SPS - 4.24
MIGS-32	Gene calling method	Prodigal 1.4, GenePRIMP
	NCBI project ID	60455
	INSDC ID	CP002775
	Genbank Date of Release	October 12, 2011
	GOLD ID	Gc01776
	Project relevance	Biocontrol, Agriculture

### Growth conditions and DNA isolation

*S. plymuthica* AS13 was grown in Luria Broth (LB) medium at 28 °C until early stationary phase. The DNA was extracted from the cells by using a standard CTAB protocol for bacterial genomic DNA isolation that is available at JGI [32].

### Genome sequencing and assembly

The genome of *S. plymuthica* AS13 was sequenced using a combination of Illumina and 454 sequencing platforms. The details of library construction and sequencing can be found at the JGI [32]. The sequence data from Illumina GAii (1,457.3 Mb) were assembled with Velvet [33] and the consensus sequence was computationally shredded into 1.5 kb overlapping fake reads. The sequencing data from 454 pyrosequencing (79.5 Mb) were assembled with Newbler and consensus sequences were computationally shredded into 2 kb overlapping fake reads. The initial draft assembly contained 86 contigs in 1 scaffold. The 454 Newbler consensus reads, the Illumina Velvet consensus reads and the read pairs in the 454 paired end library were assembled and quality assessment performed in the subsequent finishing process by using software phrap package [34-37]. Possible mis-assemblies were corrected with gapResolution [32], Dupfinisher [38], or by sequencing cloned bridging PCR fragments with subcloning. The gaps between contigs were closed by editing in the software Consed [37], by PCR and by Bubble PCR primer walks (J.-F. Chang, unpublished). Fifty one additional reactions were necessary to close gaps and to raise the quality of the finished sequence. The sequence reads from Illumina were used to correct potential base errors and increase consensus quality using the software Polisher developed at JGI [39]. The final assembly is based on 46.8 Mb of 454 draft data which provides an average 8.7 × coverage of the genome and 1,415.6 Mb of Illumina draft data which provides an average 262.2 × coverage of the genome.

### Genome annotation

The *S. plymuthica* AS13 genes were identified using Prodigal [40] as part of the genome annotation pipeline at Oak Ridge National Laboratory (ORNL), Oak Ridge, TN, USA, followed by a round of manual curation using the JGI GenePRIMP pipeline [41]. The predicted CDS were translated and used to search the National Center for Biotechnology Information (NCBI) nonredundant database, Uniport, TIGR-Fam, Pfam, PRIAM, KEGG, COG and InterPro databases. Non-coding genes and miscellaneous features were predicted using tRNAscan-SE [42], RNAmmer [43], Rfam [44], TMHMM [45], and signalP [46]. Additional gene prediction analysis and functional annotation was performed within the Integrated Microbial Genomes – Expert Review (IMG-ER) platform developed by the Joint Genome Institute, Walnut Creek, CA, USA [47].

## Genome properties

**Figure 3:**
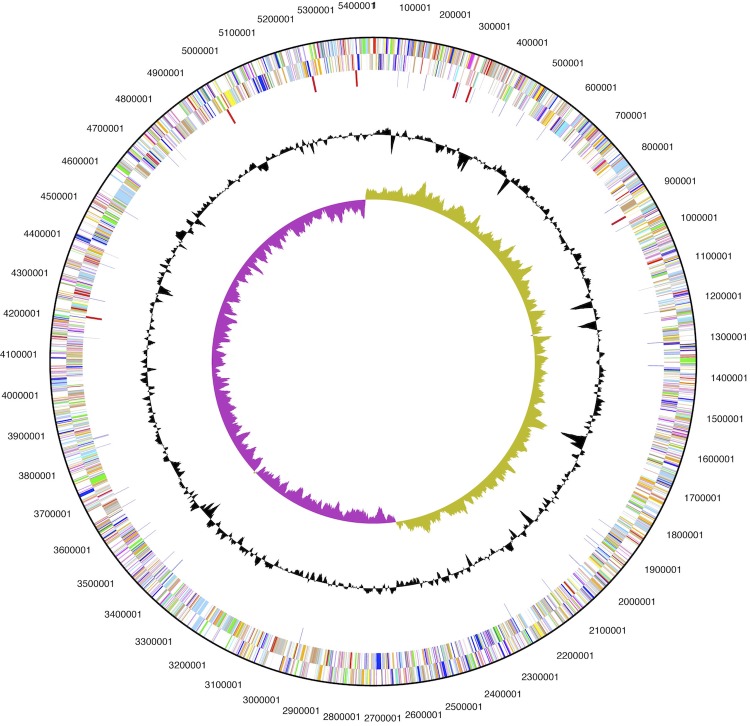
Graphical circular map of the chromosome. From outside to the center: Genes on forward strand (color by COG categories), Genes on reverse strand (color by COG categories), RNA genes (tRNAs blue, rRNAs red, other RNAs black), GC content, GC skew.

The genome of *S. plymuthica* AS13 has a single circular chromosome of 5,442,549 bp with 55.96% GC content (Table 3 and Figure 3). It has 5,139 predicted genes, of which 4,951 were assigned as protein-coding genes. Among them, most of the protein coding genes (84.41%) were functionally assigned while the remaining ones were annotated as hypothetical proteins. 112 genes were assigned as RNA genes and 76 as pseudogenes. The distribution of genes into COG functional categories is presented in Table 4.

**Table 3:** Genome statistics

**Attribute**	**Value**	**% of total^a^**
Genome size (bp)	5,442,549	100.00%
DNA Coding region (bp)	4,770,475	87.65%
DNA G+C content (bp)	3,045,680	55.96%
Total genes	5,139	100.00%
RNA genes	112	2.18%
rRNA operons	7	0.14%
Protein-coding genes	4,951	96.34%
Pseudogenes	76	1.48%
Genes in paralog clusters	112	2.18%
Genes assigned to COGs	3,805	74.04%
Genes assigned in Pfam domains	4,183	81.39%
Genes with signal peptides	676	13.15%
Genes with transmembrane helices	1,228	23.89%
CRISPR repeats	1	% of totala

a) The total is based on either the size of the genome in base pairs or the total number of protein coding genes in the annotated genome.

**Table 4:** Number of genes associated with the 25 general COG functional categories

**Code**	**Value**	**% age**	**Description**
J	201	4.27	Translation, ribosomal structure and biogenesis
A	1	0.02	RNA processing and modification
K	480	10.20	Transcription
L	161	3.42	Replication, recombination and repair
B	1	0.02	Chromatin structure and dynamics
D	37	0.79	Cell division and chromosome partitioning
Y	0	0.00	Nuclear structure
V	64	1.36	Defense mechanisms
T	187	3.97	Signal transduction mechanisms
M	265	5.63	Cell envelope biogenesis, outer membrane
N	94	2.00	Cell motility and secretion
Z	0	0.00	Cytoskeleton
W	0	0.00	Extracellular structure
U	116	2.47	Intracellular trafficking and secretion
O	153	3.25	Posttranslational modification, protein turnover, chaperones
C	272	5.78	Energy production and conversion
G	424	9.01	Carbohydrate transport and metabolism
E	470	9.99	Amino acid transport and metabolism
F	106	2.25	Nucleotide transport and metabolism
H	185	3.93	Coenzyme metabolism
I	135	2.87	Lipid metabolism
P	285	6.06	Inorganic ion transport and metabolism
Q	133	2.83	Secondary metabolite biosynthesis, transport and catabolism
R	537	11.41	General function prediction only
S	398	8.46	Function unknown
-	918	17.86	Not in COG
